# Magnetic resonance imaging in naso-oropharyngeal carcinoma: role of texture analysis in the assessment of response to radiochemotherapy, a preliminary study

**DOI:** 10.1007/s11547-023-01653-2

**Published:** 2023-06-19

**Authors:** Eleonora Bicci, Cosimo Nardi, Leonardo Calamandrei, Eleonora Barcali, Michele Pietragalla, Linda Calistri, Isacco Desideri, Francesco Mungai, Luigi Bonasera, Vittorio Miele

**Affiliations:** 1 Department of Experimental and Clinical Biomedical Sciences, Radiodiagnostic Unit n. 2, University of Florence - Azienda Ospedaliero-Universitaria Careggi, Largo Brambilla 3, 50134 Florence, Italy; 2 Department of Information Engineering, University of Florence, 50139 Florence, Italy; 3 Radiation Oncology, University of Florence - Azienda Ospedaliero-Universitaria Careggi, Largo Brambilla 3, 50134 Florence, Italy; 4 Department of Radiology, University of Florence - Azienda Ospedaliero-Universitaria Careggi, Largo Brambilla 3, 50134 Florence, Italy

**Keywords:** Naso-oropharyngeal carcinoma, Head and neck, Texture analysis, Magnetic resonance imaging, Radiochemotherapy

## Abstract

**Objective:**

Identifying MRI texture parameters able to distinguish inflammation, fibrosis, and residual cancer in patients with naso-oropharynx carcinoma after radiochemotherapy (RT-CHT).

**Material and methods:**

In this single-centre, observational, retrospective study, texture analysis was performed on ADC maps and post-gadolinium T1 images of patients with histological diagnosis of naso-oropharyngeal carcinoma treated with RT-CHT. An initial cohort of 99 patients was selected; 57 of them were later excluded. The final cohort of 42 patients was divided into 3 groups (inflammation, fibrosis, and residual cancer) according to MRI, 18F-FDG-PET/CT performed 3–4 months after RT-CHT, and biopsy. Pre-RT-CHT lesions and the corresponding anatomic area post-RT-CHT were segmented with 3D slicer software from which 107 textural features were derived. T-Student and Wilcoxon signed-rank tests were performed, and features with *p*-value < 0.01 were considered statistically significant. Cut-off values—obtained by ROC curves—to discriminate post-RT-CHT non-tumoural changes from residual cancer were calculated for the parameters statistically associated to the diseased status at follow-up.

**Results:**

Two features—Energy and Grey Level Non-Uniformity—were statistically significant on T1 images in the comparison between ‘positive’ (residual cancer) and ‘negative’ patients (inflammation and fibrosis). Energy was also found to be statistically significant in both patients with fibrosis and residual cancer. Grey Level Non-Uniformity was significant in the differentiation between residual cancer and inflammation. Five features were statistically significant on ADC maps in the differentiation between ‘positive’ and ‘negative’ patients. The reduction in values of such features between pre- and post-RT-CHT was correlated with a good response to therapy.

**Conclusions:**

Texture analysis on post-gadolinium T1 images and ADC maps can differentiate residual cancer from fibrosis and inflammation in early follow-up of naso-oropharyngeal carcinoma treated with RT-CHT.

## Introduction

Head and Neck neoplasms are the seventh most common type of cancer worldwide [1]. The most frequent histological type is squamous cell carcinoma (SCC) including oropharyngeal carcinoma (OPC) that is the most common neoplasm and nasopharyngeal carcinoma (NPC) [2]. Alcohol and smoking are the risk factors most associated with SCC [3]. Infection by oncogenic variants of human papilloma virus (HPV) is another very important risk factor [4] and HPV status has a deep impact on risk profiling [1]. HPV positive SCC is typical of young patients with high socio-economic status and it usually has an earlier diagnosis with favourable prognosis [5–8]. As a testament to this difference, HPV positivity has become part of the TNM classification [5]. In case of NPC, non-keratinizing squamous cell carcinoma (type II NPC) and undifferentiated carcinoma (type III NPC) are more common in geographical areas where NPC is endemic—mainly north Africa and east and south-east Asia—and often carry a favourable prognosis [2]. While the role of HPV in the pathogenesis of NPC is not clear, Epstein-Barr virus infection appears to be strongly correlated [2]. Magnetic Resonance Imaging (MRI) is the reference imaging technique for the evaluation of SCC due to its better soft tissue visualization than Computed Tomography (CT) [8, 9]. For this reason, MRI is also recommended for staging by the American Joint Committee on Cancer [2, 10–12]. Chemoradiotherapy (RT-CHT) is one of the cornerstones for treatment of both early and locally advanced forms of NPC, although outcome is very heterogeneous [6, 13–17]. Therefore, it is desirable to develop markers for the prediction and early detection of failure or response to treatment [14]. To this end, other techniques have been used alongside traditional MRI, namely dynamic contrast-enhanced perfusion imaging (DCE-PWI) and diffusion weighted imaging (DWI) [18]. Although they have been shown to aid in both prediction and evaluation of response to RT-CHT for head and neck SCC [19], the distinction between fibrosis, inflammation, and residual cancer still proves challenging both at presentation and during post-treatment follow-up [20].

The “texture of an image” can be defined as the complex structural weave of pixels that are part of it [21]. The process of “texture analysis” studies and breaks down recurring patterns and sub-patterns that can be identified and further elaborated into quantitative “textural features” [3]. The features can then be individually studied and integrated with the results of traditional techniques of image analysis to further investigate the underlying pathology, thus helping infer more information on the patient status [3, 13, 22].

Texture analysis has been previously employed on both CT and MRI as an innovative tool to better stratify tumour phenotype in the head and neck district, mainly by non-invasively evaluating HPV status of the lesion [14, 22–27] showing promising results. The present study represents an innovative and original application of texture analysis techniques to MRI imaging, specifically ADC maps and CE-T1, of OPC and NPC to identify textural parameters that may differentiate fibrosis and inflammation from residual cancer after RT-CHT.

## Materials and methods

### Patients’ selection

This was a single-centre, observational, retrospective study. Between January 2014 and January 2022, all patients with histological diagnosis of OPC or NPC who underwent MRI both before and after RT-CHT in the radiology department of the Careggi Hospital of Florence (Italy) were selected by searching our Picture Archiving and Communication System (PACS).

This study was approved by the research ethics committee (protocol n. 21800_oss) and informed written consent was obtained from all individual participants included in the study.

Inclusion criteria:

- Patients aged more than 18 years,
- Histological diagnosis of OPC or NPC confirmed by biopsy,
- Patients underwent RT-CHT,
- MRI examinations performed at 3–4 months follow-up after ending RT-CHT, with the same MRI scanner to make the sample as homogeneous as possible.

**Fig. 1:**
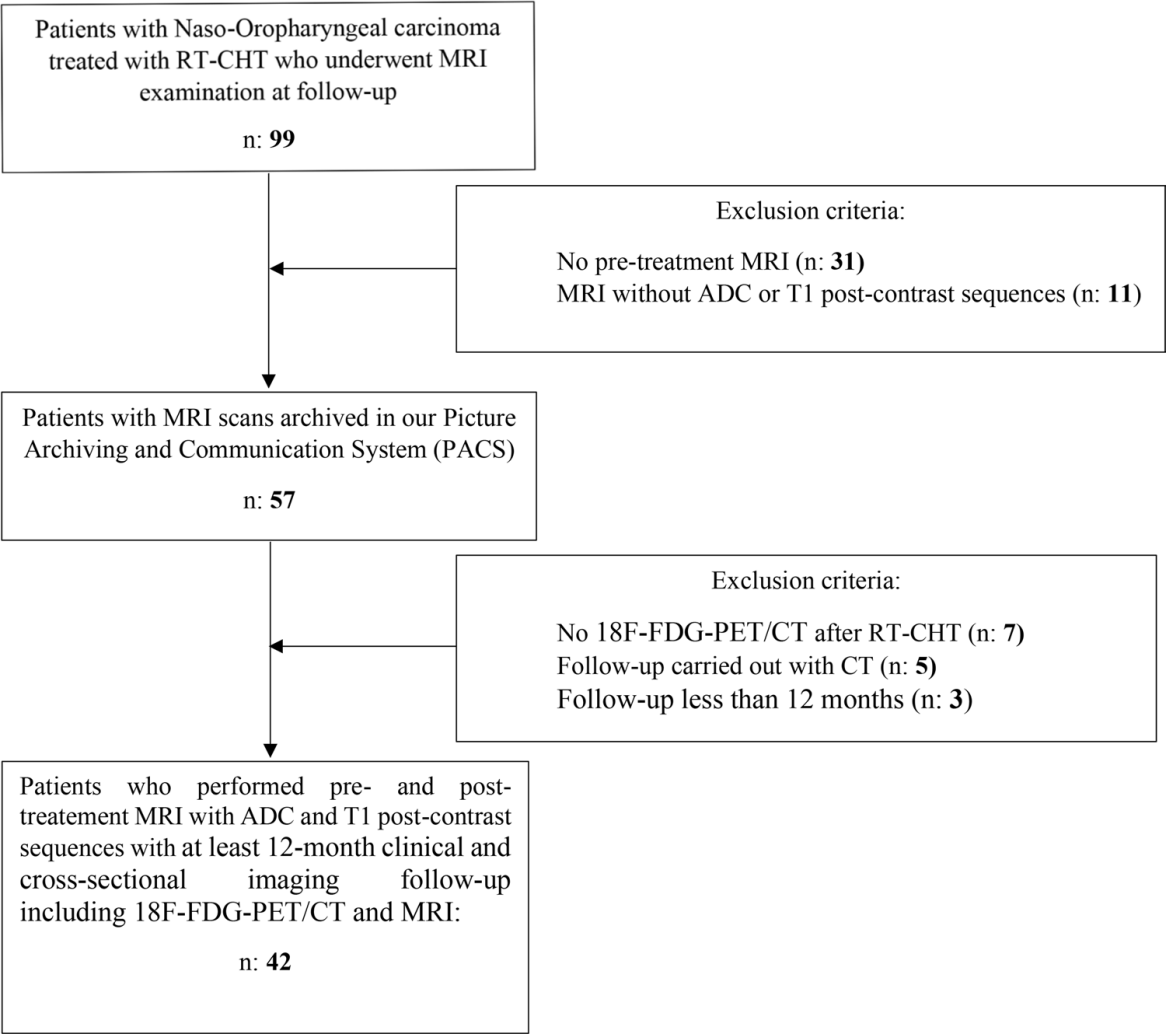
Workflow of patients’ selection

Exclusion criteria:

- No pre-treatment MRI,
- No apparent diffusion coefficient (ADC) maps and post-contrast T1 MRI sequences,
- No 18F-FDG-PET/CT in the follow-up carried out 3–4 months after RT-CHT,
- Clinical and cross-sectional imaging follow-up including 18F-FDG-PET/CT and MRI shorter than 12 months,
- Previous head and neck radiotherapy treatment,
- Previous head and neck surgery,

The workflow of patients’ selection is shown in Fig. 1. The initial population included 99 patients of whom 42 had no pre-treatment MRI performed in our hospital or were not studied with ADC and/or post-contrast T1 MRI sequences. Seven patients had no 18F-FDG-PET/CT after RT-CHT, five patients continued their follow-up with CT examinations instead of MRI and three patients had a follow-up less than 12 months.

### Images acquisitions and analysis

MRI examinations were performed with 1.5 T Magnetom Aera (Siemens Healthcare, Erlangen, Germany). The study protocol involved unenhanced scans including sagittal fat saturated T1- and T2- weighted sampling sequences with axial, coronal, and sagittal multiplanar reconstructions; axial T2-weighted turbo spin echo; axial fat saturated echo-planar DWI spectral attenuated inversion recovery (SPAIR) and ADC maps. Enhanced scans performed after intravenous gadolinium chelates contrast agent injection (gadobutrol, 1 mL/10 kg, flow 3 mL/sec followed by 20 mL saline flush) consisted of an axial T1-weighted turbo spin echo and axial T1-weighted volumetric interpolated breath-hold examination (VIBE) Dixon. Further information about acquisition protocol is detailed in Table 6 in Appendix 1.

A radiologist with 5 years’ experience in head and neck imaging (E.B.) visually segmented the entire volume of both the primary tumour and the corresponding anatomic area on post-therapy images. Such segmentations were performed on ADC maps and VIBE CE-T1 sequences by the volumetric ROI (region of interest) function within the open-source software 3DSlicer (software version 4.10.2, https://www.slicer.org/). ROIs were delineated slice-by-slice for each patient by the radiologists. Textural features extraction was carried out by means of SlicerRadiomics tool. A total of 107 features from the PyRadiomics lists—an open-source python package for the extraction of radiomics data from medical images—were selected, belonging to First Order, 3D Shape Based, Gray Level Co-occurrence Matrix, Gray Level Size Zone Matrix, Gray Level Run Length Matrix, Neighbouring Gray Tone Difference Matrix, and Gray Level Dependence Matrix classes.

### Division into groups based on imaging and histological examination

**Fig. 2:**
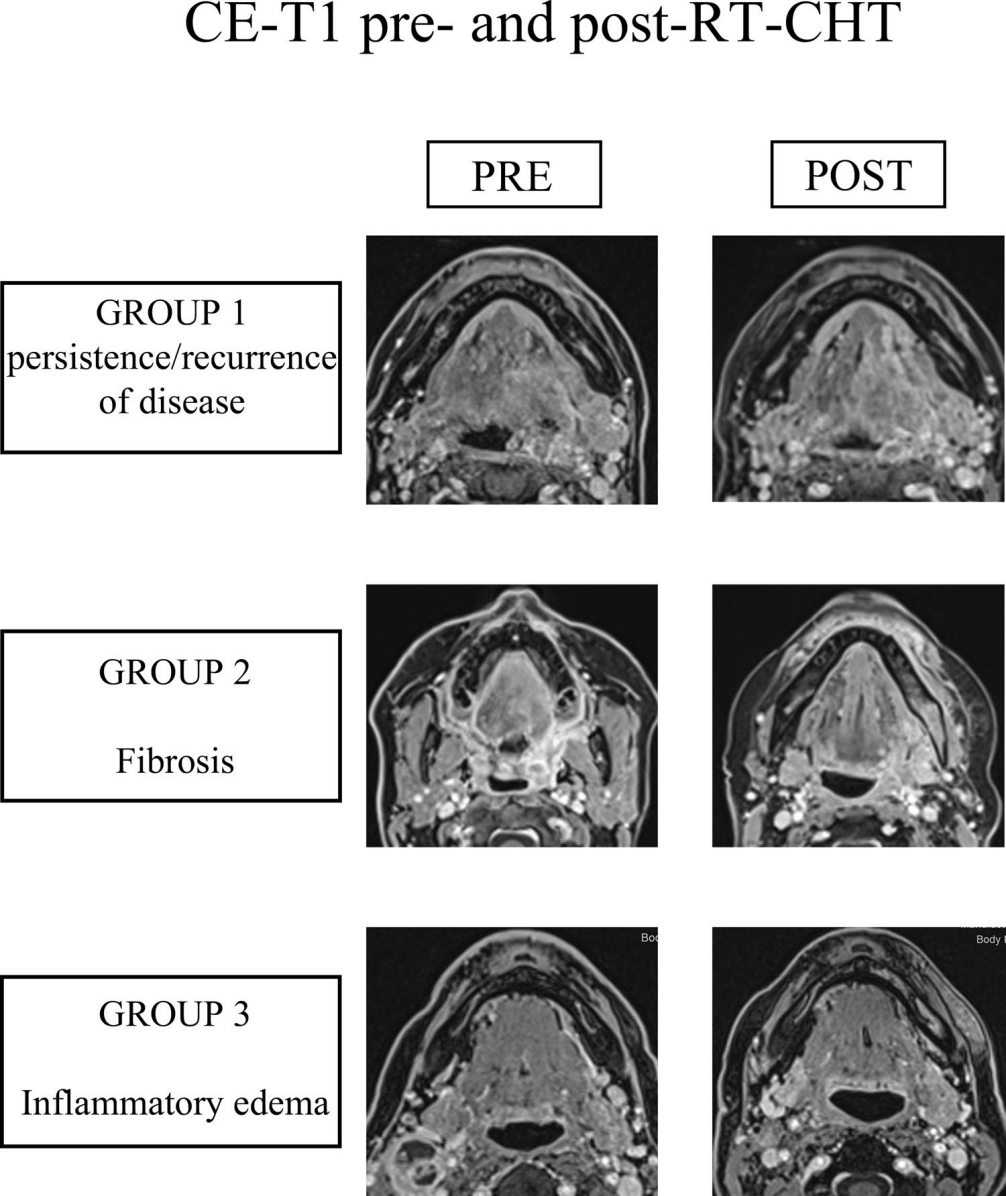
Post-RT-CHT changes in patients belonging to different groups

We first divided the patients into two groups based on MRI, 18F-FDG-PET/CT and/or biopsy results. ‘Positive’ group (group 1) included patients with persistence or recurrence of disease, whereas ‘negative’ group included patients with no residual cancer, but with tissue signal alterations on MRI compatible with post-treatment changes such as fibrosis and inflammation (groups 2 and 3, respectively). Exemplary post-RT-CHT findings from each group are shown in Fig. 2. More specifically, ‘negative’ patients were subdivided in patients with fibrosis and inflammatory edema according to MRI morphologic parameters, 18F-FDG-PET/CT results and/or biopsy performed in those cases suspicious for recurrence/persistence of disease after RT-CHT. Therefore, the groups of patients were assessed separately.

*Group 1*: persistence/recurrence of disease–residual cancer—7 patients.

Masslike lesions with moderately high (intermediate) signal intensity on T2 images; restricted diffusion due to high tumoural cells density with a subsequent decrease in ADC values (1.09 ± 0.29 × 10^−3^ mm^2^/s); non-homogeneous enhancement after gadolinium with intensity signal similar to primary tumour; positivity of 18F-FDG-PET/CT; positive biopsy.

*Group 2*: fibrosis, 19 patients.

Linear or triangular alterations with very low signal intensity on T2 images—similar to or lower than muscle—for late fibrosis (post-radiotherapy scar); low ADC values in late fibrosis (0.98 ± 0.26 × 10^−3^ mm^2^/s) since the tissue is mainly composed of densely packed collagen but normally in combination with lack of diffusion restriction (hypointense signal on DWIb800 images); low enhancement after gadolinium; negative biopsy.

*Group 3*: inflammatory edema, 16 patients.

Wide ill-defined alterations with high signal intensity on T2 images for post-radiotherapy tissue reaction; hypointense signal intensity on DWIb800 images; high ADC values (1.75 ± 0.34 × 10^−3^ mm^2^/s); vivid homogeneous enhancement after gadolinium due to inflammation; positivity of 18F-FDG-PET/CT; negative biopsy.

The aforementioned tissue features and especially ADC values used in differentiating patients within the three groups were referred to a study by Ailianou et. al on the detection of post-treatment head and neck SCC [28].

### Statistical analysis

Firstly, a Shapiro–Wilk test was performed to determine the nature of the distribution of data. Parameters that showed a normal distribution were analysed using the parametric t-Student test. All parameters that did not show a normal distribution were studied with the Wilcoxon signed-rank test. These tests identified the *p*-value for each parameter investigated. Once the *p*-values were obtained, they were compared with the level of significance, thus determining if the null hypothesis—the mean difference between the coupled samples is zero—should be accepted (equals to 0) or rejected (equals to 1).

Once the most significative features (*p*-value < 0.01) were obtained, we compared both the values between the positive (persistence/recurrence of disease) and negative (fibrosis and inflammatory edema) groups and the values among the three groups separately to analyse significative changes of parameters related to the presence of residual cancer, fibrosis, or inflammation.

In order to compare the negative dataset with the positive dataset since they had a number of different patients, we randomly took small subgroups of the negatives consisting of a larger number of patients and compared them with the positive values. In this way we could evaluate the results a greater number of times in addition to studying the variations.

For the parameters that showed statistically significant association with the diseased status at follow-up, a cut-off value to discriminate post-RT-CHT non-tumoural changes with respect to residual cancer was calculated using Receiver Operating Characteristic (ROC) curve analysis. In particular, sensitivity and specificity were calculated for the entire spectrum of values and cut-off were chosen as the values with the highest sensitivity and specificity at the same time. The area under the ROC curve was considered as a measure of the overall performance of each parameter—diagnostic accuracy—to discriminate the diseased status at follow-up. Finally, we created box plots to assess whether there were significant changes in the values of the selected features between pre- and post-RT-CHT.

## Results

The final sample included 42 patients (24 women and 18 men; mean age 59 years, median age 58.5 years, range 36–81 years); 29 patients were affected by OPC (16 HPV positive, 4 HPV negative, and 9 unknowns for HPV status) and the remaining 13 patients by NPC.

On T1 images after gadolinium, two features were statistically significant in the comparison between ‘positive’ and ‘negative’ groups. These were Energy (*p*-value = 0.003), a first order feature that represents a measure of magnitude of voxel values in an image and Grey Level Non- Uniformity (*p*-value = 0.007)—belonging to Grey Level Dependence Matrix—(Table 1).

**Table 1:** Mean values and *p*-values for the most significant (*p*-value < 0.01) features in both contrast-enhanced T1 MRI (CE-T1 MRI) and apparent diffusion coefficient maps (ADC) into the groups of patients with residual cancer (positives) and patients with inflammatory edema or fibrosis (negatives)

Statistically significant Textural Features (Positives vs. Negatives)			
*CE-T1-MRI*			
Feature name	Mean value positive	Mean value negative	P-value
Energy	1.35*10^9^	3.91*10^8^	0.003
Grey level non uniformity	644.547	236.396	0.008
*ADC map*			
Large dependence emphasis	5.554	3.004	0.001
Dependence variance	1.295	0.583	0.001
Run variance	0.047	0.002	0.001
Zone variance	3.643	0.612	0.001
Large area emphasis	6.742	2.395	0.001

Post-gadolinium T1 texture parameter Energy appeared to be helpful in the characterization of the 'positive’ group (group 1) versus both the ‘negative group’ (groups 2 and 3 together) (AUC = 0.6; Cut-off = 1*10^9^; SEN = 75%; SPEC = 90%) and patients with fibrosis alone (group 2) (AUC = 0.75; Cut-off = 4*10^8^; SEN = 75%; SPEC = 90%).

Energy was also found to be statistically significant in both groups of patients with fibrosis (group 2) and residual cancer (group 1) (*p*-value = 0.018) (Table 2).

**Table 2:** Mean values and *p*-values for the most significant (*p*-value < 0.01) features in both contrast-enhanced T1 MRI (CE-T1 MRI) and Apparent diffusion coefficient maps (ADC) in the into the groups of patients with residual cancer (positives) and patients with fibrosis

Statistically significant textural features (Positives vs. Fibrosis)			
*CE-T1-MRI*			
Feature name	Mean value positive	Mean value Fibrosis	*P*-value
Energy	1.35*10^9^	6.43*10^8^	0.009
*ADC map*			
Large dependence emphasis	5.554	3.106	0.008
Dependence variance	1.295	0.612	0.001
Run variance	0.047	0.023	0.007
Zone variance	3.643	0.663	0.001
Large area emphasis	6.742	2.488	0.001

Grey Level Non-Uniformity was significant in the differentiation between residual cancer (group 1) and inflammatory edema (group 3) (*p*-value = 0.019) (Table 3).

**Table 3:** Mean values and *p*-value for the most significant (*p*-value < 0.01) features in both contrast-enhanced T1 MRI (CE-T1 MRI) and apparent diffusion coefficient maps (ADC) into the groups of patients with residual cancer (positives) and patients with inflammatory edema

Statistically significant Textural Features (Positives vs. Inflammation)			
*CE-T1-MRI*			
Feature name	Mean value positive	Mean value inflammation	*P*-value
Grey level non uniformity	644.547	143.398	0.002
*ADC map*			
Large dependence emphasis	5.554	2.844	0.002
Dependence variance	1.295	0.536	0.002
Run variance	0.047	0.023	0.002
Zone variance	3.643	0.532	0.005
Large area emphasis	6.742	2.253	0.006

Grey Level non-Uniformity was therefore the other significant feature which we have found in the differentiation between ‘negative’ and ‘positive’ patients on post-gadolinium T1 images (AUC = 0.74; Cut-off = 511; SEN = 75%; SPEC = 96%) and especially between patients with inflammatory edema and residual cancer (AUC = 0.78; Cut-off = 510; SEN = 76%; SPEC = 92%).

On ADC maps, five features were statistically significant both in the differentiation between ‘positive’ and ‘negative’ groups (Table 1) and more specifically in the differentiation between residual cancer and fibrosis (Table 2) or inflammatory edema (Table 3). These were Large Dependence Emphasis, Dependence Variance, Run Variance, Zone Variance, and Large Area Emphasis.

In the characterization of the 'positive’ group (group 1) versus the ‘negative group’ (groups 2 and 3 together), Dependence Variance (AUC = 0.76; Cut-off = 1.04; SEN = 75%; SPEC = 96%) and Large Dependence Emphasis (AUC = 0.77; Cut-off = 4.38; SEN = 75%; SPEC = 96%) were two significant features belonging to Grey Level Dependence Matrix.

Run Variance (AUC = 0.76; Cut-off = 0.04; SEN = 75%; SPEC = 96%) belonged to Grey Level Run Length Matrix and represented the variance in runs for the run lengths, therefore the number of pixels that had the same grey level value in an image.

Zone Variance (AUC = 0.76; Cut-off = 1.43; SEN = 75%; SPEC = 96%) and Large Area Emphasis (AUC = 0.76; Cut-off = 3.90; SEN = 75%; SPEC = 96%) belonged to Grey Level Size Zone.

**Fig. 3:**
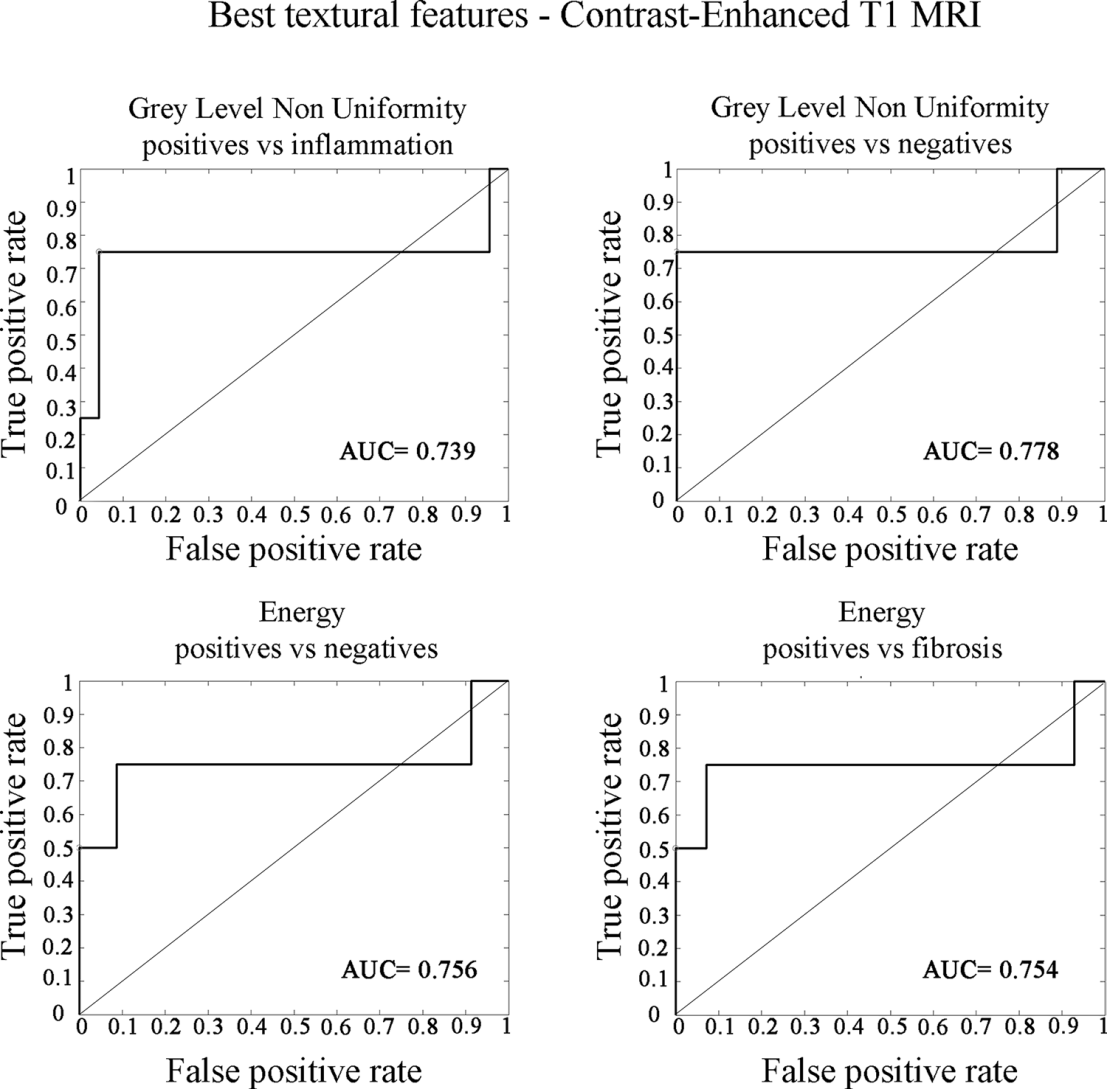
Receiver Operating Characteristic (ROC) curve analysis of post-gadolinium T1 images. Upper part of the image: correlation of Grey Level Non-Uniformity feature between patients with residual cancer (positives) and inflammatory edema (inflammation) and between positives and negatives patients. Negative patients are patients with tissue alterations characterized by inflammatory edema or fibrosis. Lower part of the image: correlation of Energy feature between positive and negative patients and between patients with residual cancer (positives) and residual fibrosis (fibrosis). The features showed an Area under the curve (AUC) between 0.7 and 0.8, that is indicative of an acceptable level of discrimination between the two groups of patients

**Fig. 4:**
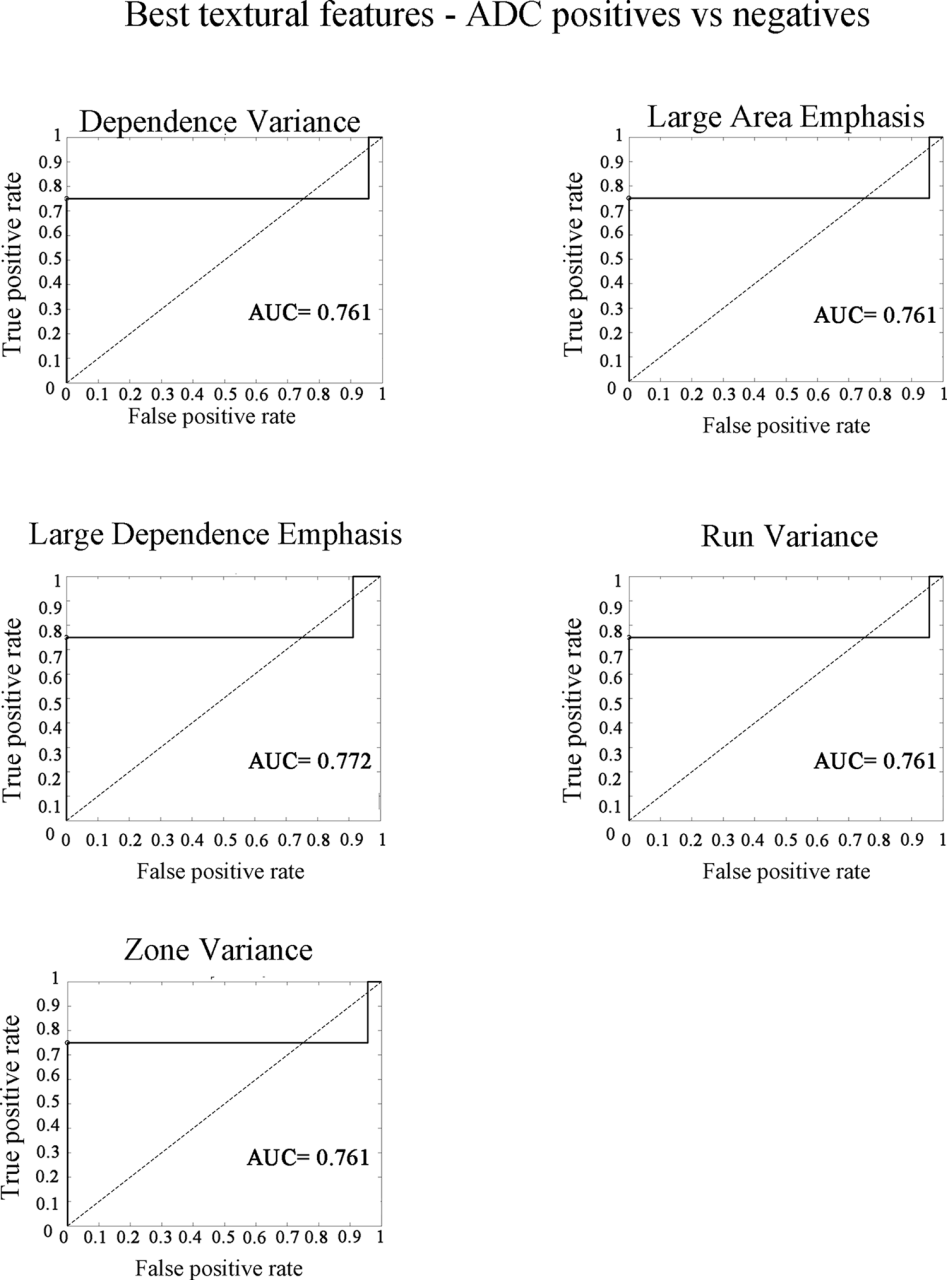
Receiver Operating Characteristic (ROC) curve analysis of Apparent Diffusion Coefficient (ADC) map images of the five features resulted to be statistically significant (Dependence variance, Large Area Emphasis, Large Dependence Emphasis, Run Variance, Zone Variance). The features showed an Area under the curve (AUC) between 0.7 and 0.8, that is indicative of an acceptable level of discrimination between the’positive’ and ‘negative’ patients

Area under the receiver operating characteristic (ROC) curve (AUC), cut-off values, sensitivity (SEN), specificity (SPE), and diagnostic accuracy for each statistically significant texture parameter with AUC ≥ 0.5 are shown in Tables 4 and 5, respectively. ROC curves are illustrated in Figs. 3 and 4.

**Table 4:** Cut-off values, sensitivity, and specificity for the most significant (*p*-value < 0,01) features among the three groups of patients (Positives vs. Negatives, Positives vs. Fibrosis, Positives vs. Inflammation) in both contrast-enhanced T1 MRI (CE-T1 MRI) and Apparent diffusion coefficient maps (ADC)

MRI sequence	Group	Feature	AUC	Cutoff	Sensitivity	Specificity
CE-T1	Positives versus Negatives	Energy	0.63	1*10^9^	0.75	0.90
CE-T1	Positives versus Fibrosis	Energy	0.75	4*10^8^	0.75	0.90
CE-T1	Positives versus Negatives	Grey level non uniformity	0.74	511	0.75	0.96
CE-T1	Positives versus Inflammation	Grey level non uniformity	0.78	510	0.76	0.92
ADC	Positives versus Negatives	Dependence variance	0.76	1.04	0.75	0.96
ADC	Positives versus Negatives	Large area emphasis	0.76	3.90	0.75	0.96
ADC	Positives versus Negatives	Large dependence emphasis	0.77	4.38	0.75	0.96
ADC	Positives versus Negatives	Run variance	0.76	0.04	0.75	0.96
ADC	Positives versus Negatives	Zone variance	0.76	1.43	0.75	0.96
ADC	Positives versus Fibrosis	Dependence variance	0.77	1.04	0.75	0.93
ADC	Positives versus Fibrosis	Large Area Emphasis	0.77	3.90	0.75	0.93
ADC	Positives versus Fibrosis	Large dependence emphasis	0.77	5.19	0.76	0.96
ADC	Positives versus Fibrosis	Run variance	0.77	0.04	0.73	0.94
ADC	Positives versus Fibrosis	Zone variance	0.77	2.81	0.76	0.98
ADC	Positives versus Inflammation	Dependence variance	0.75	5.86	0.75	1.00
ADC	Positives versus Inflammation	Large area emphasis	0.75	5.86	0.76	0.96
ADC	Positives versus Inflammation	Large dependence emphasis	0.75	5.86	0.74	0.89
ADC	Positives versus Inflammation	Run variance	0.75	5.86	0.70	0.85
ADC	Positives versus Inflammation	Zone variance	0.75	2.82	0.72	0.86

**Table 5:** Cut-off values and diagnostic accuracy for the most significant (*p*-value < 0.01) features in both contrast-enhanced T1 MRI (CE-T1 MRI) and apparent diffusion coefficient maps (ADC) for the distinction between patients with residual cancer and patients with inflammatory edema or fibrosis. Values higher than the cut-off are indicative of residual cancer, whereas values lower than the cut-off are indicative of non-tumoral changes characterized by inflammatory edema or fibrosis

Diagnostic accuracy of significant textural features		
*CE-T1-MRI*		
*Feature name*	*Cut-off*	*Accuracy*
Energy	1*10^9^	85%
Grey level non-uniformity	4*10^8^	92%
*ADC map*		
Dependence variance	1.03	92%
Large area emphasis	3.90	92%
Large dependence emphasis	4.38	92%
Run variance	0.03	92%
Zone variance	1.42	92%

**Fig. 5:**
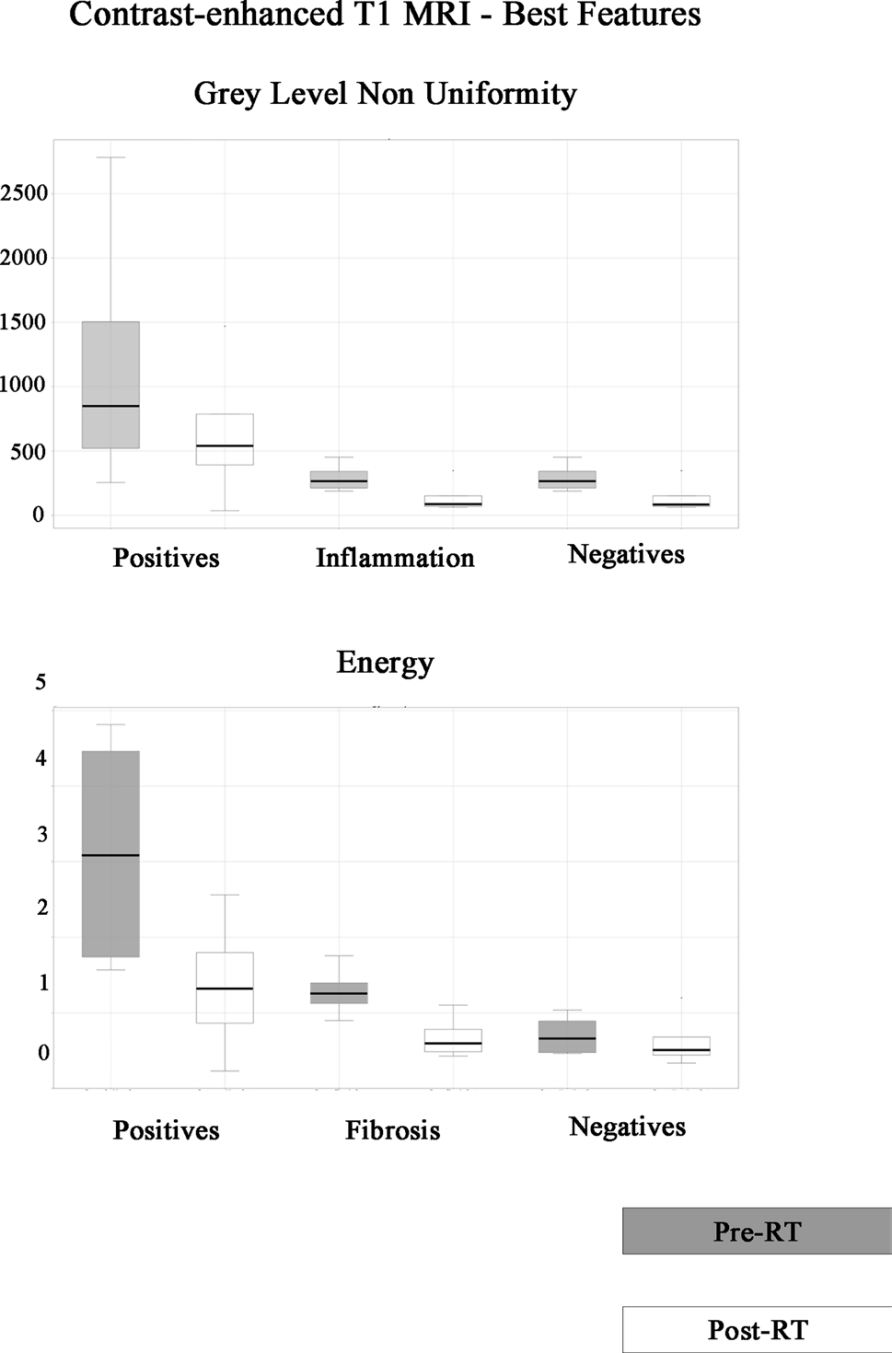
Distribution of Grey Level Non Uniformity texture feature on pre and post radiochemotherapy post-gadolinium T1 images in patients with residual cancer (positives), inflammatory edema only and both inflammatory edema and fibrosis (negatives). Distribution of Energy feature on pre and post radiochemotherapy post-gadolinium T1 images in positives, fibrosis only and negatives patients. A marked reduction in values between pre and post radiochemotherapy features was found in patients with fibrosis only, inflammatory edema only and no residual cancer (negatives including both inflammatory edema and fibrosis). In contrast, a smaller reduction in values can be observed in positive patients for persistence/recurrence of disease, especially for Grey level non Uniformity

The comparison of the distribution of feature values between pre- and post-RT-CHT on T1 images after gadolinium and ADC maps was significantly different in ‘positive’ and ‘negative’ groups and also in patients with fibrosis and inflammatory edema. The distribution of Grey Level Non-Uniformity texture parameter on post-gadolinium T1 images both in pre and post RT-CHT (Fig. 5) showed how the ‘negative group’ and especially patients with inflammatory edema had a marked reduction in feature values between pre- and post-therapy.

In contrast, the same reduction in values in patients positive for persistence/recurrence of disease was not observed. The same distribution of values was found for Energy feature on post-gadolinium T1 images analysed pre and post RT-CHT in fibrosis (group 2) and ‘negative’ groups (groups 2 and 3 together), with only a limited reduction of Energy value in patients with residual cancer (group 1).

**Fig. 6:**
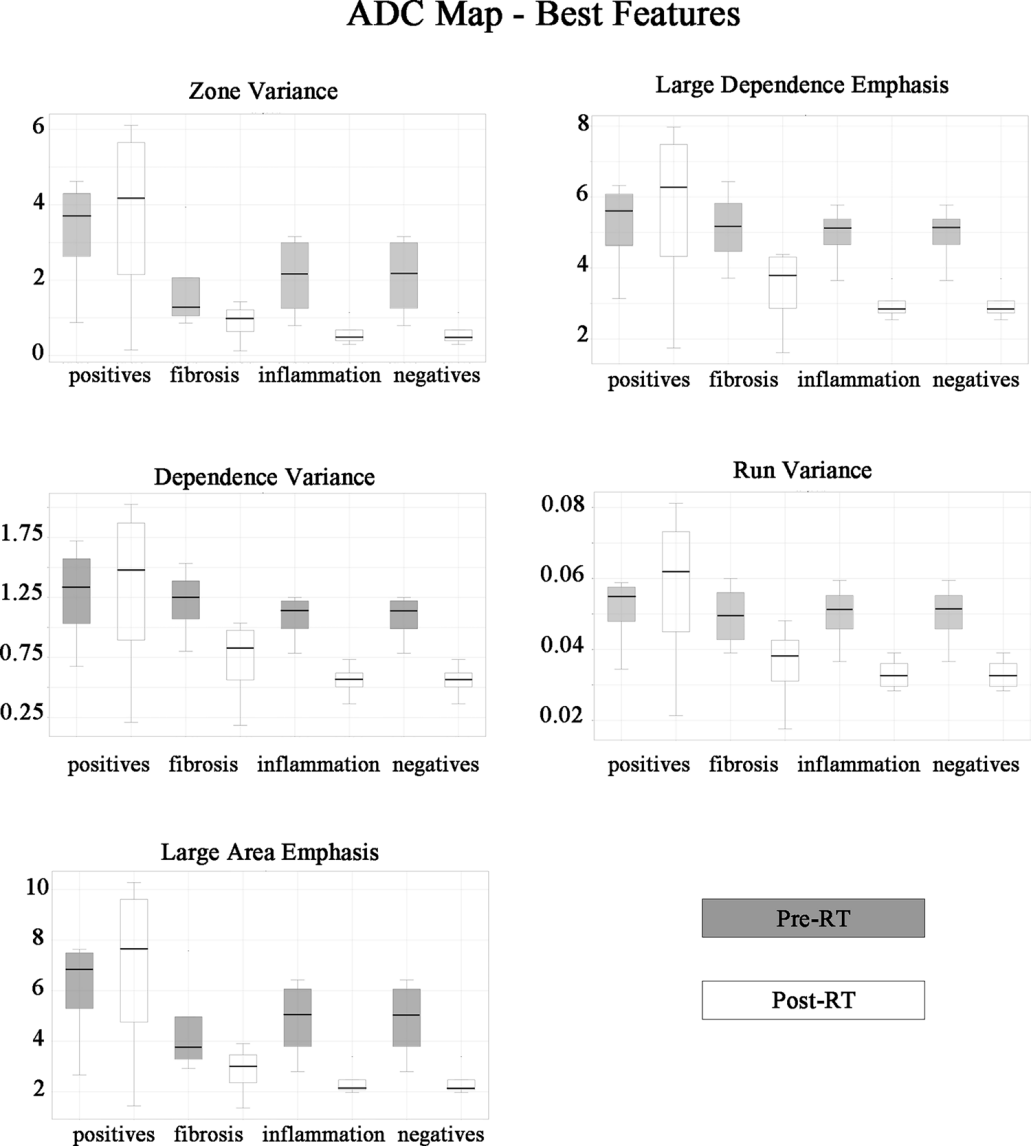
Distribution of textural parameters on pre and post radiochemotherapy Apparent diffusion Coefficient map images in patients with residual cancer (positives), inflammatory edema only, fibrosis only and both inflammatory edema and fibrosis (negatives). A marked reduction in values between pre and post radiochemotherapy features in fibrosis only, inflammatory edema only and with no residual cancer patients (negative group including both inflammatory edema and fibrosis) can be observed. In contrast, no reduction in values in positive patients for persistence/recurrence of disease can be observed

Furthermore, the distribution of texture parameters on ADC maps in the ‘negative’ group—both fibrosis and inflammation groups—showed a high reduction in values of all features analysed pre-RT-CHT compared to post RT-CHT. In contrast, there was not the same trend of reduction in values between pre and post RT-CHT in patients with residual cancer (Fig. 6).

No significant feature was found capable of differentiating fibrosis from inflammation on VIBE CE-T1 sequences and ADC maps.

## Discussion

The current study represented the first challenge to use texture analysis on MRI images to discriminate residual cancer from non-tumoral changes in patients with OPC and NPC who underwent exclusive RT-CHT. Two texture features on post-gadolinium T1 images (Energy and Grey Level Non-Uniformity) and five texture features on ADC maps (Large Dependence Emphasis, Dependence Variance, Run Variance, Zone Variance, and Large Area Emphasis) resulted to be statistically significant in the detection of tumour persistence/recurrence. The substantial reduction in values of such features between pre- and post-RT-CHT was correlated with a good response to therapy and the development of non-tumoral radiotherapy-induced changes, whereas the stability of high values after RT-CHT was associated with the presence of residual cancer.

A previous study by Cozzi et al. [29] analysed how texture analysis features correlate with the local control after RT-CHT in Head and Neck tumours. They used CT as imaging technique and found maximum value, volume, and small-zone high gray-level emphasis as statistically significant predictors of response to therapy. The crucial goal of our study was the identification of features capable of differentiating tumour persistence/recurrence from fibrosis and inflammatory edema both on post-gadolinium T1 images and ADC maps. We decided to analyse T1 images since cancers typically have different characteristics with respect to fibrosis and inflammation after gadolinium administration. Cancers usually show inhomogeneous and intermediate enhancement that represents a middle way between active inflammation and fibrous tissue. Generally, tumour tissue tends to be heavily enhanced and similar to that of the primary tumour because of its intrinsic hypervascularity with intra-lesional inhomogeneity due to necrosis foci or irregularities, whereas inflammatory tissue and non-newly formed fibrotic tissue have a more homogeneous enhancement and low contrast agent uptake, respectively [30]. Post-gadolinium T1 texture parameter Energy appeared to be helpful in the characterization of the 'positive’ group (group 1) versus both the ‘negative group’ (groups 2 and 3 together) and patients with fibrosis alone (group 2). Energy had much higher values on post-gadolinium T1 images (Tables 1 and 2) in patients with persistent/recurrence of disease than patients with non-tumoural changes and especially in case of non-newly formed fibrosis (Table 2) since tumour tissue had greater enhancement. On the contrary, limited or lacking gadolinium spread in fibrotic tissue resulting in lower image voxel values and lower Energy values accordingly. In two studies conducted by Park et al. [26] and Tomita et al. [31, 32], Energy was a substantial feature in the differentiation between metastatic and benign lymph nodes, underlining how it may be useful in discriminating neoplastic tissue from non-pathological tissue. Grey Level non-Uniformity was the other significant feature which we have found in the differentiation between ‘negative’ and ‘positive’ patients on post-gadolinium T1 images and especially between patients with inflammatory edema and residual cancer. Low values of Grey Level non-Uniformity correlated with a greater similarity in intensity values. But even in this case the values of this feature were much higher in patients with residual cancer (Tables 1 and 3) than post-RT-CHT non-tumoural changes. As mentioned for Energy, Grey Level non-Uniformity correlated with the presence of tumour tissue characterized by high cellularity and vivid uptake of contrast agent.

In recent years, DWI and ADC map values have been becoming crucial tools for enhancing diagnosis of both malignant and benign diseases [33]. Unfortunately, their use is not always conclusive since overlaps in values can be found. The human eye can only assess ADC maps qualitatively by comparing them with the surrounding tissue map values [34]. However, texture analysis can examine the nature of the grey level transitions and the spatial relations of the pixels included in the regions of interest [4]. We exploited texture analysis on ADC maps for their correlation with intra-tumoural changes in cellularity, angiogenesis, extravascular extracellular matrix, and areas of necrosis [35]. We looked for a possible correlation between texture parameters and MRI, 18F-FDG-PET/CT, and histopathology. More precisely, we investigated the role of texture analysis on post-gadolinium and DWI images related to the high structural heterogeneity within an area subject to treatment for cancer—hypervascularisation, necrosis, and high cellularity—and its potential to discern between tumour and tissue changes such as inflammation or fibrosis. On ADC maps, in the comparison between ‘positive’ and ‘negative’ groups, five features resulted as statistically significant in the differentiation between persistence/recurrence of disease and post-RT-CHT non-tumoral changes (Table 4). In particular, all five selected features were found to have higher values in patients with residual cancer than ‘negative’ ones. Higher values were also found in patients with residual cancer (group 1) in comparison with the group of fibrosis (group 2) and inflammatory edema (group 3) each of them taken individually. In the characterization of the 'positive’ group (group 1) versus the ‘negative group’ (groups 2 and 3 together), Dependence Variance and Large Dependence Emphasis were two significant features belonging to Grey Level Dependence Matrix. Dependence Variance measured the variance in dependence size in the image. The higher the value, the more variation there was within the image. In the current study, higher values of these features meant greater textural non-homogeneity. They were mainly found in tumoral tissues compared to fibrotic or inflammatory alterations because of the presence of necrotic foci in residual cancer.

Run Variance belonged to Grey Level Run Length Matrix and represented the variance in runs for the run lengths, therefore the number of pixels that had the same grey level value in an image. By increasing the value, as in the case of residual cancer, there was more variation within the image, which supported what was already explained for the previous features and especially the necrotic tissue and intra-lesional non-homogeneity.

Zone Variance and Large Area Emphasis belonged to Grey Level Size Zone. Zone Variance represented the number of connected voxels that have the same grey level intensity and measured the variance in zone size values for the zone, with higher values in a more inhomogeneous tissue as tumour is. Large Area Emphasis was a measure of the distribution of large area size zones with greater values indicative of more large size zones and more coarse textures, demonstrating the typical inhomogeneity of tumour tissues. Both features showed higher values in positive patients, being a representation of the structural inhomogeneity typical of tumoral tissue.

All significant above-mentioned features showed good diagnostic accuracy ranging from 85 to 92% (Table 5). When compared to literature data relating to the diagnostic accuracy of CE-T1-MRI and ADC maps in treated OPC and NPC [39, 40], cut-off values attained by texture analysis for the differentiation between residual cancer (group 1) and non-tumoral changes (groups 2 and 3 together) yielded higher specificity (96% vs. 86% for ADC, 96% vs. 82% for CE-T1-MRI) and lower sensitivity (75% vs. 89% for ADC; 75% vs. 84% for CE-T1-MRI). Our results suggested that the addition of texture analysis should lead to improved diagnostic performance and more accurate distinction between tumour persistence and fibrotic or inflammatory alterations in post-treatment patients.

In clinical practice, texture analysis should be integrated with other MR parameters such as T2, enhancement T1 and DWI to better characterize the tissue investigated.

In our opinion, the role of texture analysis on MRI with ADC maps and post-gadolinium T1 images will become clinically significant when full data processing is performed in the shortest possible time and implemented in software systems where radiologists are used to visualise images. It took us 15 extra minutes to examine the texture features of each patient. We decided to use an open-source software so that everyone could access it in clinical practice. This aspect should be underlined because the use of the same open and free datasets with defined parameters for extracted features will guarantee a greater reproducibility of studies. The most difficult challenge for a widespread implementation of texture analysis processes is the lack of a standardised method due to the large number of procedures for the texture feature extraction, resulting in a large amount of information that is hardly comparable [24]. Due to the large variability of many factors in the execution of examinations, such as MRI devices with their respective parameters, filters, acquisition sequences, post-processing algorithms, as well as the type of software used (free and non-free commercially available or custom-made in-house applications), attaining standardised and reproducible results is still rather difficult [17, 26, 36].

A first limitation of our study was the performance of examinations carried out via an MRI-unit only. Although this was an advantage to make the sample as homogeneous as possible, it was currently a disadvantage for the lack of reproducibility of results. According to the literature, the study by Mackin et al. [37] underlined how variations in the acquisition parameters and reconstruction techniques may affect features extractions. As regards future developments, it would be interesting to repeat the current study on different datasets of patients, with more patients and MRI-units involved, to confirm or not the association of the features we have found with persistence/recurrence of disease or post-RT-CHT non-tumoural changes. Another limitation was the relative low sample size. Nevertheless, most papers on texture analysis of OPC and NPC did not include both pre- and post-RT-CHT MRI examinations [8, 13, 14, 29, 38–40]. In addition, the small number of patients with residual cancer (7 individuals) had to be related to the well-known excellent response to RT-CHT of OPC—especially when HPV positive—and NPC. Moreover, it is known that HPV + and HPV − SCC differ in radiological imaging and prognosis, thus representing a possible bias in the current study [27].

Then, our single-centre results cannot be generalized until more evidence is gathered. Future studies should collect a larger amount of persistence/recurrence of pharyngeal tumour to strengthen our findings. Further analyses should also be conducted to identify cut-off values that may differentiate residual cancer from non-tumoural tissue changes induced by RT-CHT to attain better clinical decision making and improve the diagnostic accuracy.

## Conclusions

Texture analysis on post-gadolinium T1 images and ADC maps was a useful tool in the early follow-up of OPC and NPC treated with RT-CHT to differentiate residual cancer from fibrosis and inflammatory edema. The substantial reduction in values of some features—including Energy and Grey Level non-Uniformity on post-gadolinium T1 images and Large Dependence Emphasis, Dependence Variance, Run Variance, Zone Variance, and Large Area Emphasis on ADC maps—between pre- and post-RT-CHT was correlated with a good response to therapy and the development of RT-CHT-induced changes, whereas no or little variation of values of such features after RT-CHT was associated with residual cancer. No feature enabled a clear differentiation between fibrosis and inflammation.

## Appendix 1

See Table 6

**Table 6:** MRI acquisition parameters. Unenhanced scans included T1 and T2 sampling perfection with application-optimized contrasts using different flip angle evolution (SPACE) sequences with axial, coronal, and sagittal multiplanar reconstructions; axial T2 turbo spin echo; axial fat saturated echo-planar DWI spectral attenuated inversion recovery (SPAIR) with two b-values (b 50- 800 s/mm2) and ADC maps. Enhanced scans carried out after intravenous gadolinium contrast agent (gadobutrol, 1 mL/10 kg, flow 3 mL/sec followed by 20 mL saline flush) consisted of an axial T1 turbo spin echo and axial T1 volumetric interpolated breath-hold examination (VIBE) Dixon

Sequence	Contrast agent	Repetition time (ms)	Echo time (ms)	Slice thickness (mm)	Interslice gap (mm)	Field of view (mm)	Matrix	Acceleration factor	Number of signal averaged	Band width (Hz/Px)	Acquisition time (min:sec)	Voxel size
SPACE T1-w Sagittal	pre	500	7.2	0.9	–	229 × 229	230 × 256	2	1.4	630	5:47	0.9 × 0.9 × 0.9
SPACE T2-w Sagittal Fat-Sat	pre	3000	380	0.9	–	229 × 229	230 × 256	2	1.4	698	5:56	0.9 × 0.9 × 0.9
TSE T2-w Axial	pre	5050	117	3	0.9	210 × 190	261 × 484	2	3	191	2:23	0.5 × 0.5 × 3.0
SPAIR EPI-DWI Axial (b 50/800 s/mm2)	pre	4100	55	3	0.9	240 × 240	102 × 128	3	1	1608	3:09	1.6 × 1.6 × 3.0
VIBE T1-w DCE-PWI Axial; FA 5°, 15°	pre	4.65	1.66	3.5	0.7	250 × 226	139 × 132	3	1	390	1:04	1.3 × 1.3 × 3.5
TSE T1-w Axial	post	440	17	3	0.9	200 × 181	384 × 384	3	3	200	2:31	0.5 × 0.5 × 3.0
VIBE Dixon Axial	post	10	2.4	0.9	0.18	225 × 225	212 × 256	–	1	340	4:37	0.9 × 0.9 × 0.9
VIBE T1-w DCE-PWI Axial; FA 30°	post	4.65	1.66	3.5	0.7	250 × 226	139 × 132	3	1	300	4:17	1.3 × 1.3 × 3.5
